# Emergency Liver Resection with Staplers for Spontaneous Liver Haemorrhage in a Patient Receiving Anticoagulant Therapy

**DOI:** 10.1155/2013/204046

**Published:** 2013-07-14

**Authors:** Koray Kutlutürk, Vural Soyer, Abuzer Dirican, Bulent Unal, Cemalettin Aydin, Cuneyt Kayaalp, Sezai Yilmaz

**Affiliations:** Department of General Surgery, Liver Transplantation Institute, Inonu University, 44280 Malatya, Turkey

## Abstract

*Introduction*. Emergency liver resection during active bleeding in a patient who takes anticoagulant therapy is a complicated and high-risk surgery. *Aim*. We described a technique that is combination of staplers, total hepatic vascular occlusion, and hemostatic agent (TachoSil) application for safe and quick hepatectomy. *Patient and Method*. A 72-year-old woman who uses warfarin regularly due to valvuloplasty admitted emergency unit with abdominal pain and shock. At admission, her hemoglobin, hematocrit, and INR values were 5.2 g/dL, 14.9%, and 6.7, respectively. Radiologic evaluation revealed abdominal free fluid and a liver lesion on segments V, VI, and VII. Emergency laparotomy was required. There was an active bleeding from a liver hematoma that could not be controlled by packing, and an urgent hepatic resection was required. Under total hepatic vascular occlusion, segments V, VI, and VII were resected with endoscopic nonvascular staplers. Cut surface of the liver was coagulated with bipolar cautery and covered with a hemostatic material. *Results*. Hepatectomy took six minutes, and the duration of surgery was 80 minutes. There was no complication and no transfusion required after surgery, and the patient was discharged on 8th day, uneventfully. *Conclusion*. Emergency hepatectomy with staplers, under vascular control with hemostatic agents, provided a rapid and safe surgery.

## 1. Introduction

Nowadays, urgent liver resection is rarely required due to the successes of interventional radiology and conservative liver surgery. However, urgent liver resection can still be necessary with a significant morbidity and mortality when the other methods failed [1]. Here, we described a case that the patient who had anticoagulant therapy was admitted to the hospital in shock due to a spontaneous liver hemorrhage. The patient was treated by urgent hepatectomy with staplers under total vascular occlusion and with the help of local hemostatic agents.

## 2. Case Presentation

**Figure 1:**
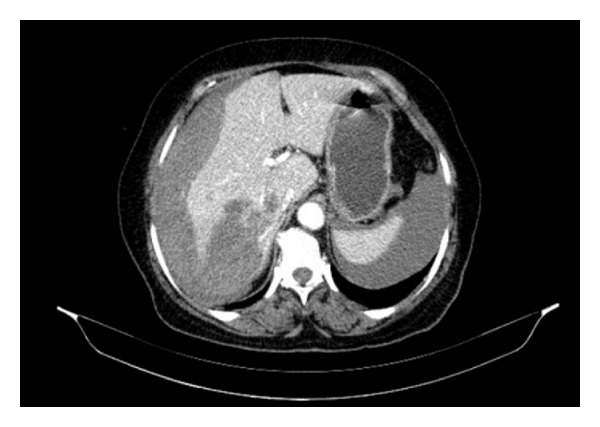
Preoperative abdominal computed tomography.

**Figure 2:**
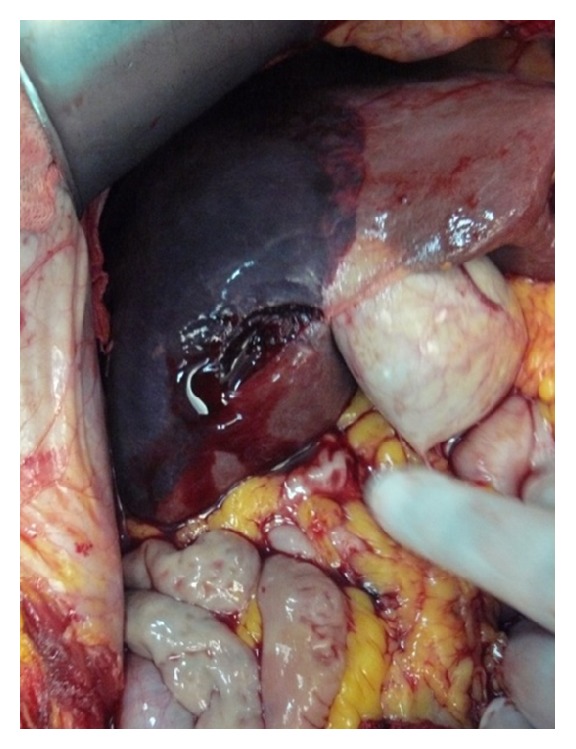
Bleeding hematoma at segment V.

**Figure 3:**
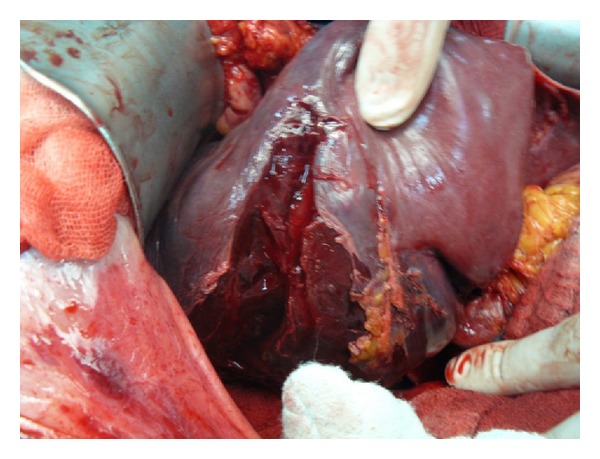
Bleeding lacerated liver at segments VI and VII.

**Figure 4:**
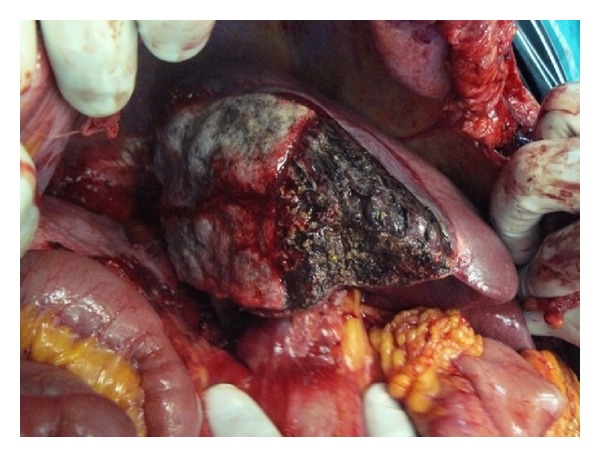
Coagulated and topical hemostatic covered cut surface of the liver.

A 72-year-old woman admitted to emergency unit with acute abdominal pain. She had a history of open heart surgery (valvuloplasty), and she was taking warfarin regularly. There was no trauma history. Her blood pressure, pulse, and respiratory rates were 78/42 mmHg, 112/min, and 28/min, respectively. There was a generalized abdominal pain that was more apparent on the right upper quadrant. Ultrasound demonstrated free abdominal fluid and an irregular lesion at the right liver lobe. Computed tomography revealed abdominal free fluid and a liver lesion on segments V, VI, and VII (Figure 1). Liver pathology was not clearly diagnosed, but it was reported as a possibly ruptured hemangioma. Her hemoglobin, hematocrit, and INR values were 5.2 g/dL, 14.9%, and 6,7, respectively. Her general condition was deteriorated during examinations despite intravenous fluid supports, blood transfusions, and inotropic infusions. Emergency laparotomy was required and 1500 mL blood was aspirated from the abdomen. At the first sight, there was large subcapsular liver hematoma, which was still bleeding from a 4 cm length tear on segment V of the liver (Figure 2). We packed the liver and waited for cessation of the bleeding, but it was still going on particularly from the posterior part of the liver. Right liver was mobilized from the posterior attachments, and there was a large subcapsular hematoma on the right posterior sector of the liver. Blood was coming out from a tear of the liver capsule. The liver capsule was removed to see the origin of bleeding. Active hemorrhage was coming from the liver parenchyma through the two deep lacerations at segment VI and VII (Figure 3), and there was a palpable focal lesion in the liver at the same location. By Pringle maneuver, we blocked the blood inflow of the liver and packed the liver again, but prolonged INR failed the success of packing. We decided an urgent resection of the bleeding part of the liver including the mass. Under total hepatic vascular occlusion, segments V, VI, and VII were resected with endoscopic staplers (Covidien endostaplers, US). We used 10 nonvascular (blue-cartilage) staplers because there was no vascular stapler at that time in the operating room. Before applying staplers, we created tunnels in the liver parenchyma by a long clamp. Hepatectomy time took almost six minutes, and the cut surface of the liver was coagulated with bipolar cautery and covered with a hemostatic material (TachoSil, Takeda Company, Zurich). Total vascular clamping was released, and the cut surface of the liver was pressed with moisturized gauzes for a few minutes. There was no bleeding (Figure 4), and surgery was ended within total 80 minutes. During surgery, total six packs of erythrocyte and four packs of fresh frozen plasma were transfused. Postoperative period was uneventful, and the patient did not require any extra blood transfusion. There was no complication, and the patient was discharged on day eight. Pathological result was a surprise for us that the mass was belonged to *Fasciola hepatica*. After surgery the patient was consulted with the infectious diseases unit, and Triclabendazol was recommended.

## 3. Discussion

Massive hemoperitoneum due to nontraumatic rupture of the liver is a rare condition. There is usually an underlying liver disease such as a benign (hemangioma) or a malignant liver neoplasm (hepatocellular carcinoma). More rarely, spontaneous liver hemorrhage may develop depending on the underlying disorder of hemostasis in case of cirrhotic patients or in patients taking anticoagulants such as warfarin. In this case, *Fasciola hepatica* was an unexpected liver histopathology. It was reported that *Fasciola hepatica* can cause liver abscesses or metastasis-like masses in the liver, but it has not been reported as a cause of spontaneous liver hemorrhage itself [2, 3]. Here, *Fasciola hepatica* might have been a trigger role at the beginning of liver hemorrhage, and the disordered hemostasis might be the aggravating reason of the hemorrhage. It was difficult to find out the main reason, but we believe that the main reason of liver hemorrhage was the severe underlying hemostatic disorder, and *Fasciola hepatica* was an incidental diagnostic finding. 

If there is a spontaneous subcapsular liver hematoma without hemoperitoneum, a conservative, nonoperative approach should be considered. Conservative treatments can be used even if there was an ongoing hemorrhage as well. If hemorrhage is not stopped by conservative treatments or in case of hemodynamic instability or existence of aggravating risk of hemorrhage due to impaired hemostasis, the indications of surgical intervention should be taken care. In this case, all three of the above-mentioned indications for surgery were present. At surgery, packing, suturing, or coagulations are the most frequently used methods for liver hemostasis. It is well known that emergency hepatic resection has a significant morbidity and mortality [1]. Even if elective hepatic resections, the operative blood loss and the number of blood transfusions are the main determinants of perioperative morbidity and mortality. In emergency conditions, more blood loss and more transfusion requirements make the hepatectomy more complicated, and the morbidities such as bleeding, liver failure, bile leakage, sepsis, and mortalities are expected even more.

We tried to find out the amount of blood loss of this hepatectomy technique. It was not easy because of the previous hemoperitoneum and ongoing hemorrhage. However, we estimated it indirectly. At the beginning of surgery the hemoglobin and hematocrit levels of the patient were 5.4 g/dL and 14.9%, respectively. During surgery, we aspirated 1500 mL free blood from the abdomen and transfused six packs of erytrocyte suspension. At the end of surgery the hemoglobin and hematocrit levels were 13.3 g/dL and 38.3%, respectively, and we did not transfuse any more blood product in the postoperative period. It is well known that one unit of red blood cell suspension increases the value of hemoglobin in of 1 g/dL and increases hematocrit value of 3% [4]. We can say that our hepatectomy technique resulted in minimal blood loss. Total hepatic vascular occlusion using staplers and topical hemostatic agents all helped us to achieve a bloodless surgery in this case. 

Vascular clamping techniques during hepatectomy are well-known methods. Pringle maneuver as an inflow blocking method is used very frequently, and total hepatic vascular occlusion is preferred less frequently during liver resections [5]. When compared Pringle maneuver alone with the total hepatic vascular occlusion (inflow and outflow blockages), blood loss was reported less in the later one [6, 7]. There are two disadvantages of total hepatic vascular occlusion; it causes severe hypotension, and it is a time-consuming procedure [6, 7]. Increasing central venous pressure before total hepatic vascular occlusion prevents hypotension, and increasing experience on liver surgery prevents time consuming. 

Transection of the liver parenchyma with the stapler came up in the 1990s [8]. Using vascular staplers was recommended as quicker and safer method with less blood loss when compared with clamp-crushing technique [9, 10]. Today, some centers use this technique as a routine procedure during hepatic resections [10]. In this case, transection of liver parenchyma with staplers also shortened the duration of total vascular clamping. Here, we used nonvascular staplers due to the absence of vascular staplers in an obligatory condition. However, this did not cause any bleeding or any other problem. 

Topical hemostatic agents are used on cut surface of liver to provide hemostasis and reduce the bile leakage and intra-abdominal collections. Thus, the amount of postoperative drainage and necessity for blood transfusion decreased. Length of hospital stay decreases as a consequence of those advantages [11]. After applying TachoSil to the cut surface of liver, oozing stopped immediately, there was no need for blood transfusion in the postoperative period, and no bile leakage was observed.

Combination of using of staplers under total hepatic vascular occlusion and applying topical potent hemostatic materials during an urgent liver surgery in an impaired hemostatic condition provided quick, safe, and effective hepatectomy.
